# Dual mechanism of chromatin remodeling in the common shrew sex trivalent (XY _1_Y _2_)

**DOI:** 10.3897/CompCytogen.v11i4.13870

**Published:** 2017-11-03

**Authors:** Sergey N. Matveevsky, Svetlana V. Pavlova, Maret M. Atsaeva, Jeremy B. Searle, Oxana L. Kolomiets

**Affiliations:** 1 N.I. Vavilov Institute of General Genetics, Russian Academy of Sciences, Gubkin str. 3, Moscow 119991, Russia; 2 A.N. Severtsov Institute of Ecology and Evolution, Russian Academy of Sciences, Leninsky pr. 33, Moscow 119071, Russia; 3 Chechen State University, A. Sheripov str. 32, Grozny 364051, Chechen Republic, Russia; 4 Department of Ecology and Evolutionary Biology, Corson Hall, Cornell University, Ithaca, NY 14853, USA

**Keywords:** Sex body, MSCI, synaptonemal complex, γH2AFX, ATR, SUMO-1, ubiH2A, *Sorex araneus*

## Abstract

Here we focus on the XY_1_Y_2_ condition in male common shrew *Sorex araneus* Linnaeus, 1758, applying electron microscopy and immunocytochemistry for a comprehensive analysis of structure, synapsis and behaviour of the sex trivalent in pachytene spermatocytes. The pachytene sex trivalent consists of three distinct parts: short and long synaptic SC fragments (between the X and Y_1_ and between the X and Y_2_, respectively) and a long asynaptic region of the X in-between. Chromatin inactivation was revealed in the XY_1_ synaptic region, the asynaptic region of the X and a very small asynaptic part of the Y_2_. This inactive part of the sex trivalent, that we named the ‘head’, forms a typical sex body and is located at the periphery of the meiotic nucleus at mid pachytene. The second part or ‘tail’, a long region of synapsis between the X and Y_2_ chromosomes, is directed from the periphery into the nucleus. Based on the distribution patterns of four proteins involved in chromatin inactivation, we propose a model of meiotic silencing in shrew sex chromosomes. Thus, we conclude that pachytene sex chromosomes are structurally and functionally two different chromatin domains with specific nuclear topology: the peripheral inactivated ‘true’ sex chromosome regions (part of the X and the Y_1_) and more centrally located transcriptionally active autosomal segments (part of the X and the Y_2_).

## Introduction

At first meiotic prophase, the male sex chromosomes in mammals form a specific heterochromatic nuclear domain (Solari 1974; Handel 2004). The structure and behaviour of the sex bivalent changes from zygotene to late diplotene. In the majority of mammal species the processes of pairing and synapsis of the X and Y chromosomes at zygotene occurs later than the same processes in autosomes. At early and mid pachytene the sex bivalent is usually located in the centre of the meiotic nucleus. At mid pachytene the sex chromosomes become shorter due to condensation and homologous regions of the X and Y are completely paired (Burgoyne 1982). Recombination nodules appear only in the short pseudoautosomal region (PAR) of the sex bivalent. In many mammals irregular thickenings may occur at asynaptic sites of axial elements of the sex bivalent. After that the sex bivalent gradually moves from the centre of the nucleus to its periphery and forms a so-called XY or sex body (Solari 1974).

The chromatin of the sex chromosomes transforms into an inactive condition and this chromatin remodelling process is known as meiotic sex chromosome inactivation (MSCI) (McKee and Handel 1993; Turner et al. 2000). MSCI is the process whereby unsynapsed regions of the sex chromosomes undergo transcriptional silencing (Lifschytz and Lindsley 1972; Handel and Hunt 1992; Turner et al. 2002, 2007); this is a case of MSUC (meiotic silencing of unsynapsed chromatin) (Schimenti 2005). The asynaptic chromatin undergoes inactivation by incorporation and modification of specific proteins (Burgoyne et al. 2009). First, BRCA1 (breast cancer 1) accumulates in non-synaptic areas of the sex chromosomes, which starts the process of phosphokinase ATR (ataxia telangiectasia- and RAD3-related) recruitment and then there is ATR-dependent phosphorylation of the γH2AFX (phosphorylated (Ser139) histone 2 A.X) histone (Turner et al. 2004). At early pachytene, ubiH2A (ubiquitinated histone H2A), SUMO-1 (small ubiquitin-related modifier-1) and other proteins are incorporated into the asynaptic chromatin of the sex chromosomes (Baarends et al. 2005). Such modification of chromatin decreases its transcriptional activity as confirmed using Cot-1 RNA FISH and RNA polymerase type II immunolocalisation (Turner et al. 2005; Baarends et al. 2005). Thus, the chromatin of the sex body is inactive.

MSCI has been well studied for the normal male sex chromosome system in mammals (XY), but there are few data on this process for multiple sex chromosome systems.

Translocation between the X and an autosome results in the formation of multiple sex chromosomes (XY_1_Y_2_; where the X is a product of a translocation between the ‘true’ X and an autosome, Y_1_ is the ‘true’ Y and Y_2_ is the autosome). The XY_1_Y_2_ condition has been demonstrated in insects (Jacobs 2003), fish (Centofante et al. 2006; de Oliveira et al. 2008) and, in particular, among mammals – including marsupials: greater bilby *Macrotis lagotis* (Sharp 1982), and placentals: Indian muntjac *Muntiacus muntjak* (Artiodactyla, Fronicke and Schertan 1997), red brocket deer *Mazama americana* (Artiodactyla, Aquino et al. 2013), big fruit-eating bat *Artibeus lituratus* (Chiroptera, Solari and Pigozzi 1994), short-tailed fruit bat *Carollia perspicillata* (Chiroptera, Noronha et al. 2009), delicate mouse *Salinomys delicates* (Rodentia, Lanzone et al. 2011), Sahel gerbil *Taterillus arenarius* and Senegal gerbil *Taterillus pygargus* (Rodentia, Ratomponirina et al. 1986; Volobouev and Granjon 1996) and others (see reviews by Fredga 1970; Sharman 1991; and Yoshida and Kitano 2012). An XY_1_Y_2_ sex chromosome system also characterises species of shrews (small insectivores) belonging to the *Sorex araneus* group (Eulipotyphla; Hausser et al. 1985), including the Eurasian common shrew *Sorex araneus* Linnaeus, 1758 which is a model system for evolutionary cytogenetics with numerous Robertsonian autosomal variants as well as the XY_1_Y_2_ condition (Searle and Wójcik 1998).

**Figure 1.**
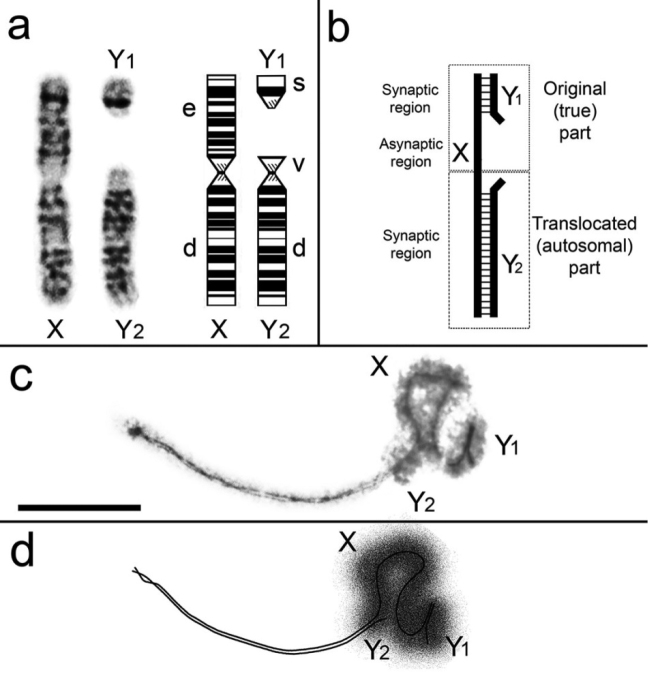
**a** G-banded sex chromosomes in the male common shrew (left) and ideogram with chromosome arms labelled according to the alphabetic nomenclature of Searle et al. (1991) **b** Schematic diagram of the shrew pachytene sex trivalent, based on Pack et al. (1993) and our data **c** Electron micrograph of a shrew sex trivalent, XY_1_Y_2_ at late pachytene. The true X region and the Y_1_ are surrounded by electron-dense material. Scale bar: 5µm. **d** Diagram of the XY_1_Y_2_ configuration as represented in Fig. 1c.

The XY_1_Y_2_ condition in the common shrew arises from a tandem fusion between an autosome and the true X chromosome (Sharman 1956, 1991; Fredga 1970; Searle et al. 1991) (Fig. 1a). Although the observation of a meiotic sex trivalent was part of the discovery of the XY_1_Y_2_ condition in the common shrew it was not until the work of Pack et al. (1993) that chromosome pairing in the XY_1_Y_2_ at meiotic prophase I was first examined. We supplemented those early observations with the discovery that the γH2AFX histone is associated with the true sex chromosome regions of the pachytene sex trivalent (Matveevsky et al. 2012).

In this paper we analyse the distribution of four transcription silencing proteins (ATR, γH2AFX, SUMO-1, ubiH2A) on the sex trivalent XY_1_Y_2_ at prophase I in common shrew spermatocytes and assess how these participate in MSCI.

## Material and methods

**Shrews.** A total of five adult males of the common shrew were collected from a locality in the vicinity of the Moscow-Neroosa chromosomal hybrid zone (near Ozyory town, Moscow Region) in April 2014, at the beginning of the breeding season. All animals were karyotyped using the method of Pavlova et al. (2008), with modifications. The trypsin-Giemsa staining technique of Král and Radjabli (1974) was used for identification of chromosome arms by G-bands, following the standard nomenclature for the *S. araneus* karyotype, which uses letters of the alphabet for chromosome arms (Searle et al. 1991).

All karyotypes were characterised by the set of invariant autosomal metacentrics *af*, *bc*, *jl* and *tu* as well as the XY_1_Y_2_ sex chromosomes system. Race-specific autosomes differed between individuals, two males had *gm*, *hi*, *kr*, *no* and *pq* metacentrics which mark the karyotype of the Moscow race. Other males had *go, hi, kr, mn* and *pq* metacentrics which characterise the Neroosa race. All shrews had the same diploid number of chromosomes (2n=21). Spermatocyte spreads were obtained from all males. All necessary national and institutional guidelines for the care and use of animals were followed.

A total of 331 cells were analysed of which 14 were prepared for electron microscopy and 317 for fluorescence microscopy. All the latter were labelled with SYCP3 (synaptonemal complex protein 3) and CREST and a proportion of cells were labelled with other antibodies (γH2AFX: 90; SUMO-1: 59; ubiH2A: 52; ATR: 32; MLH1: 74; SYCP1: 28; RNA Pol II: 10).

**Meiotic spread preparations.** Synaptonemal complex (SC) preparations were made and ﬁxed using a previously described technique (Kolomiets et al. 2010). AgNO_3_-stained slides were screened under a light microscope to select suitably spread cells. Once selected, plastic (Falcon film) circles were cut out with a diamond tip and transferred onto grids and examined in a JEM 100B electron microscope.

**Antibodies, immuncytochemistry and multistep immunostaining procedure.** Poly-L-lysine-coated slides were used for immunostaining. The slides were placed in phosphate buffer saline (PBS) and incubated overnight at 4°C with the primary antibodies diluted in antibody dilution buffer (3% bovine serum albumin - BSA, 0.05% Triton X-100 in PBS): mouse anti-MLH1 (1:50–1:100, Abcam, Cambridge, UK), rabbit polyclonal anti-SYCP1 (1:500, Abcam, Cambridge, UK), rabbit polyclonal anti-SYCP3 (1:500–1:1000, Abcam, Cambridge, UK), mouse monoclonal anti-ATR (1:200, Abcam, Cambridge, UK), human anticentromere antibody CREST (Calcinosis Raynaud’s phenomenon, Esophageal dysmotility, Sclerodactyly, and Telangiectasia) (1:500, Fitzgerald Industries International, Acton, MA, USA), mouse monoclonal anti-SUMO-1 (1:250, Zymed Laboratories, South San Francisco, CA, USA), mouse monoclonal anti-ubiquityl histone H2A (1:400, Millipore, Billerica, MA, USA), and mouse anti-phospho-histone H2AX (also known as γH2AFX) (1:1000, Abcam, Cambridge, UK).

After washing, we used the following corresponding secondary antibodies diluted in PBS: FITC-conjugated bovine anti-rabbit IgG (1:1000, Santa Cruz Biotechnology, Santa Cruz, CA, USA), goat anti-rabbit Alexa Fluor 488 (1:500, Invitrogen Corporation, Carlsbad, CA, USA), FITC-conjugated horse anti-mouse IgG (1:500, Vector Laboratories, Burlingame, CA, USA), Rodamin-conjugated chicken anti-rabbit IgG (1:400, Santa Cruz Biotechnology, Santa Cruz, CA, USA), goat anti-human Alexa Fluor 546 (1:500, Invitrogen Corporation, Carlsbad, CA, USA), goat anti-mouse Alexa Fluor 546 (1:200, 1:1000, Invitrogen Corporation, Carlsbad, CA, USA).

Immunostaining was carried out sequentially in 3 steps: 1. SYCP3/CREST (or SYCP1/MLH1); 2. ATR (or SUMO-1 or ubiH2A); 3. γH2AFX. After an each step slides were washed in PBS (6–7 times for 7–10 min) and mounted with Vectashield mounting medium containing 4,6-diamino-2-phenylIndol (DAPI) (Vector Laboratories, Burlingame, CA, USA). Slides were examined using an Axioimager D1 microscope (Carl Zeiss, Jena, Germany) equipped with an Axiocam HRm CCD camera. Images were processed using Adobe Photoshop CS3 Extended.

It should be noted that after photobleaching, bound antibodies of the first round still remain attached to the cellular structures. The more antibodies attached to the structures of interest the higher the probability that epitopes of further rounds of immunolocalisation become inaccessible. To ensure that these processes have not impacted our results, we performed control experiments for all antibodies.

**Controls.** We always conducted parallel control experiments on different slides when immunostaining was performed with a single antibody to a MSCI specific protein (double immunostaining). Our colleague Dr TM Grishaeva has conducted a bioinformatics analysis of the proteins studied. The pairwise sequence alignment of human and mouse proteins, which was performed by the COBALT program (NCBI), demonstrated high conservation of the H2AX, ubiH2A, SUMO-1, ATR and Polo II proteins. Comparison of the proteins did not reveal any problematic similarity between them. The pairwise sequence alignment of ATR and H2AX showed no amino acid sequence similarity. SUMO-1 and H2AX appeared to have 14 coincidences of amino acids, which should not affect the cross-reaction. ubiH2A and H2AX have a high level of similarity except a short sequence in the carboxyl terminus. Nevertheless, an analysis of the fluorescence intensity profile suggests a close, but not identical, picture of distribution for ubiH2A and H2AX (Matveevsky et al. 2016).

**Image analysis.** Intensity Correlation Analysis (ICA) was carried out according to Reitan et al. (2012). Scatter plots, Pearson’s coefficients (*p*_r_) and overlap correlation coefficients (*r*) were obtained using a plug-in ICA (Li et al. 2004) of ImageJ 1.45 (Rasband 1997–2016). *p*_r_ helps to evaluate the degree of correlation between the different intensities and is ranked from -1 (negative correlation) to +1 (positive correlation) (French et al. 2008). In analysing scatter plots, overlaying green and red signal resulted in a yellow signal. The more yellow in the scatter plot, the higher the level of overlap. The width of the yellow signal distribution in scatter plots corresponded to the degree of co-localisation of the fluorescence signals being compared: the wider the distribution of the signal, the higher the level of overlap of the two channels.

To evaluate the degree of co-localisation of some proteins, we have developed Fluorescent-Intensity Profiles (FIPs) using the ImageJ plug-in RGB profiler (created by Christophe Laummonerie, Jerome Mutterer, Institute de Biologie Moleculaire des Plantes, Strasbourg, France) and following Barak et al. (2010) and Fargue et al. (2013).

**Statistical analysis.** All of the data are shown as the mean values ± SD. Student’s *t*-test was performed to determine significant differences in the data. All statistical analyses were conducted using GraphPad Prism Version 5.0 (GraphPad Software, CA, USA).

## Results

### Synapsis and markers of recombination of the XY_1_Y_2_ configuration at pachytene

The sex trivalent XY_1_Y_2_ was detected in spermatocyte nuclei from the beginning of the early pachytene stage in electron micrographs. Three distinct parts are clearly visible on the sex trivalent: short and long synaptic SC segments and a long asynaptic segment of the X chromosome arranged between them. The first (short) segment of the SC (the PAR synaptic site) is formed between the true X region and the Y_1_ and is always located at the periphery of a nucleus. The second (long) segment is the SC between the translocated (autosomal) part of the X chromosome and the Y_2_ (Fig. 1); this fragment is always directed into the spermatocyte nucleus. The axial element of the X chromosome is irregularly thickened in the asynaptic region that sits between the two synaptic regions.

At the early stages of prophase I, the length of the SC between the autosomal part of the trivalent (X and Y_2_) is variable. At late zygotene and early pachytene, synapsis was observed along the entire length of the segment; while in mid pachytene desynapsis of chromosome arm *v* of Y_2_ (Fig. 1a) was detected. The length of this desynaptic segment was about 3-4% of the total length of Y_2_ (Fig. 1b).

At mid-late pachytene, a cloud of electron-dense material overlays the true sex chromosome regions which include the region of XY_1_ synapsis, the asynaptic part of the X chromosome, a short pericentromeric segment of the SC between the Х and Y_2_ and the asynaptic part of the Y_2_ (Fig. 1c–d). Thus, it is precisely this part of the XY_1_Y_2_ that takes the form of the typical sex body in male mammals.

**Figure 2.**
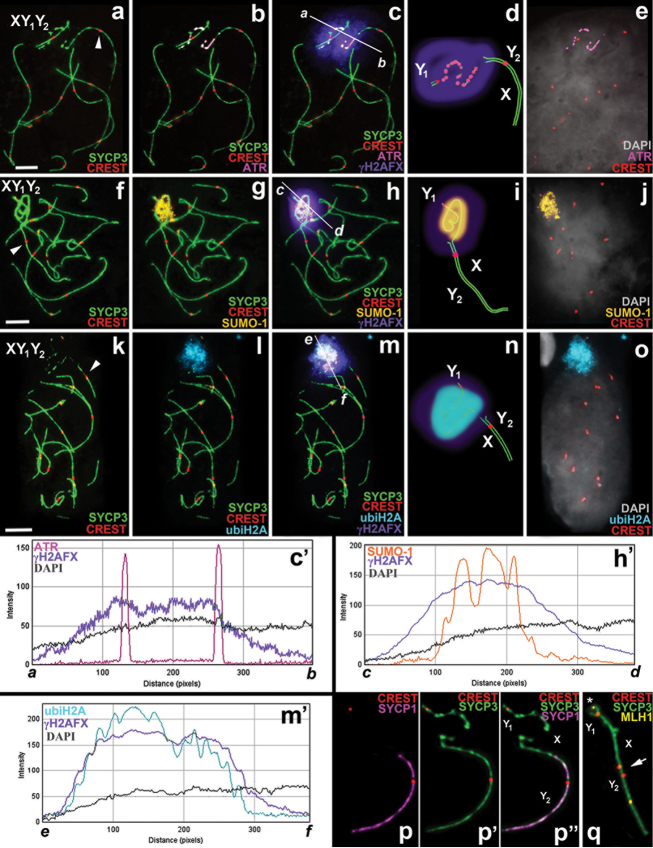
Mid-pachytene spermatocytes and male sex (XY_1_Y_2_) chromosomes of *Sorex araneus*. Bar = 5µm. The axial elements of the SC and the kinetochores were localised using anti-SYCP3 (*green*) and anti-CREST (*red*) antibodies, respectively. **a–e** ATR (*magenta*) has a discontinuous localisation within the chromatin of the true sex chromosome regions (part of the X and the Y_1_). The co-localisation of ATR, γH2AFX (*violet*), DAPI (*grey*) is shown in graph *a*-*b* (see **c** and **c**’) **f–j** SUMO-1 (*yellow*) is localised on the chromatin of true sex chromosome regions. The co-localisation of SUMO-1, γH2AFX (*violet*) and DAPI (*grey*) is shown in graph *c*-*d* (see **h** and **h**’) **k–o** ubiH2A (*cyan*) is localised on the chromatin of the true sex chromosome regions. The co-localisation of ubiH2A, γH2AFX (*violet*) and DAPI (*grey*) is shown in graph *e*-*f* (see **m** and **m**’) **d, i, n** Diagrams of the sex trivalents **p, p’, p**’’ SYCP1 (*magenta*) is located on the area of chromosome synapsis of the autosomal part of the XY_1_Y_2_ (from **a-c**) **q** XY_1_Y_2_ has two MLH1 signals (*yellow*). The MLH1 signal within the PAR synaptic site is marked by an asterisk. The arrowhead indicates the centromeres of the autosomal part of sex trivalent (part of the X and the Y_2_) which are not co-oriented with each other (*red*).

Immunostaining with antibodies against the proteins of the axial (SYCP3) and central (SYCP1) elements of the SC revealed the differences in the distribution patterns of these proteins in the sex trivalent structure. SYCP3 and SYCP1 foci were always displayed evenly and clearly on the long synaptic SC (between the Y_2_ and translocated part of the X), while the distribution foci of these proteins were either fragmentary (Fig. 2a, f, k) or completely absent in the case of SYCP1 (Fig. 2p, p’, p’’) on the short PAR synaptic fragment of SC (between the Y_1_ and the true X region).

Centromeres of the sex trivalent were detected using CREST serum. One centromere was located on the Y_1_ acrocentric and a second was seen where the X and the Y_2_ associated. Sometimes two centromeric signals were detected in this long synaptic fragment of the SC. Thus, localisation of the X and Y_2_ centromeres in the structure of the sex trivalent does not coincide.

Late recombination nodules were detected using antibodies to MLH1 (MutL homolog 1; a DNA mismatch repair protein component that is specific to these nodules). In the structure of the sex trivalent one MLH1 focus is located on the short PAR synaptic site (where the Y_1_ and the true part of X pair) and another one where the Y_2_ and translocated part of X pair (Fig. 2q).

### MSCI markers distribution in the pachytene XY_1_Y_2_

The distribution of the four transcriptional silencing proteins was analysed using immunostaining. ATR had a discontinuous localisation in the true sex chromosome regions, including a few ATR foci in the region of XY_1_ synapsis (Fig. 2a–e).

**Figure 3.**
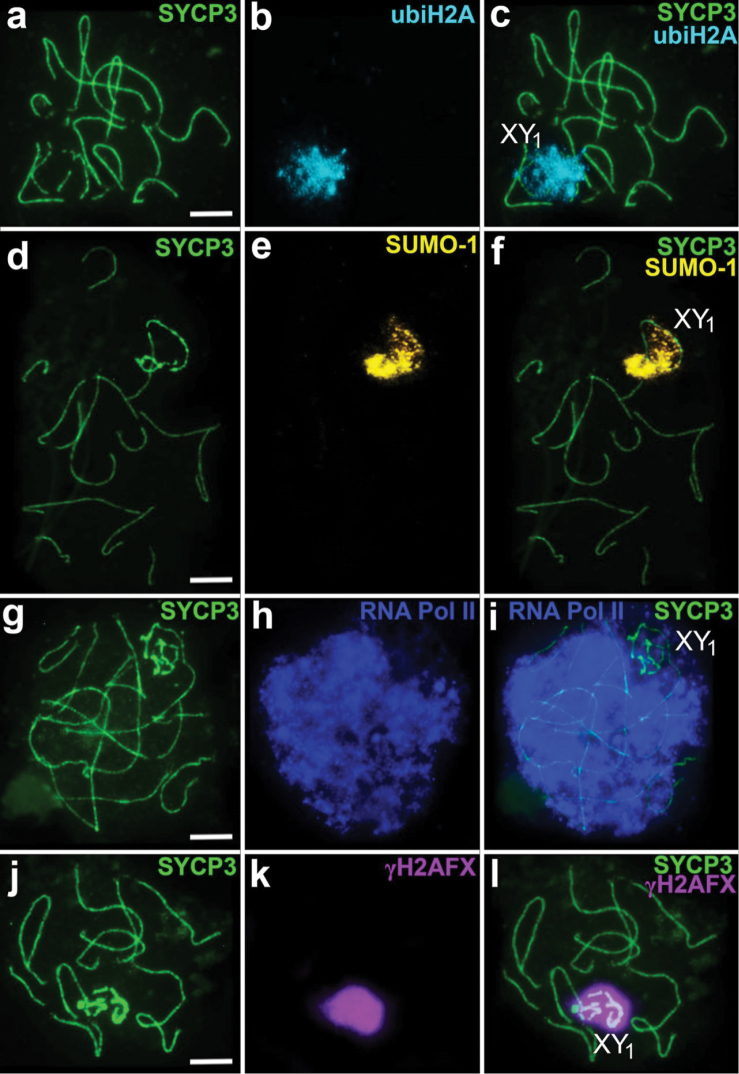
Mid-pachytene spermatocytes of *Sorex araneus*. Double immunostaining with antibodies: **a–c** anti-SYCP3 (*green*)/anti-ubiH2A (*cyan*) **d–f** anti-SYCP3 (*green*)/anti-SUMO-1 (*yellow*) **g–i** anti-SYCP3 (*green*)/anti-RNA Pol II (*blue*) **j–l** anti-SYCP3 (*green*)/anti-γH2AFX (*violet*). The true sex chromosome region is designated as XY_1_. Scale bars: 5 µm.

As a rule, as shown in our previous work on common shrews (Matveevsky et al. 2012), γH2AFX is also associated with the true sex chromosome regions within the XY_1_Y_2_, including chromatin of the asynaptic region of the X chromosome. It should be noted that the histone γH2AFX extends into the autosomal centromeric region of the XY_1_Y_2_ (Figs 2c, h, m, 3).

SUMO-1 is also localised only in the true sex chromosome regions, adjacent to the axial elements of the sex trivalent. Unlike the continuous distribution of γH2AFX, SUMO-1 has a granular pattern of localisation. The chromatin of the translocated part of XY_1_Y_2_ does not become immunostained with antibodies to the SUMO-1 (Figs 2f–j, 3).

Localisation of ubiH2A looks like an extensive cloud around the true X chromosome and Y_1_ only without extending to the autosomal part of the XY_1_Y_2_ (Figs 2k–o, 3).

**Figure 4.**
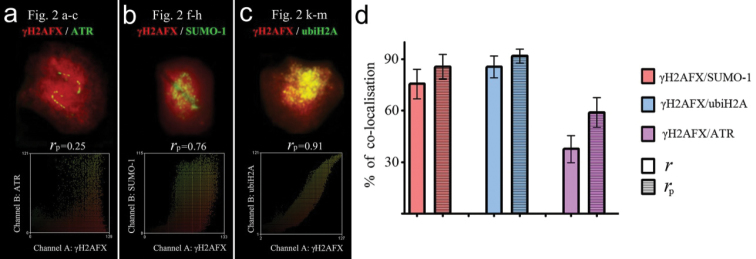
Intensity correlation analysis (ICA) represented by scatter plots showing the paired intensities of two channels (**a** γH2AFX - ATR, Fig. 2a–c **b** γH2AFX - SUMO-1, Fig. 2f–h **c** γH2AFX - ubiH2A Fig. 2k-m). *r*_p_ - Pearson correlation coefficient. See more details in the text. Degree of co-localisation for signals in sex trivalents of common shrew (**d**). On the *y*-axis, the percentage of co-localised signals are shown according to overlap correlation coefficients (*r*) and the Pearson correlation coefficient (*r*_p_).

ICA and FIPs allowed us to estimate the degree of MSCI protein co-localisation (Fig. 2c’, h’, m’). This was high for γH2AFX and ubiH2A (*r*_p_ = 0.86±0.06, *r* = 0.92±0.04; n=22) (see Fig. 4). Regarding the FIPs, the γH2AFX-signal path was similar to the ubiH2A-signal path, but slightly wider in coverage (Fig. 2m’). The degree of γH2AFX / SUMO-1 co-localisation was lower (*r*_p_ = 0.76±0.09, *r* = 0.86±0.07; n=30) (see Fig. 4). The SUMO-1 signal occupies a narrower part of the X axis and shows three peaks within the chromatin around the XY_1_ pairing region (Fig. 2h’). A significant low degree of co-localisation was found for the γH2AFX / ATR pair (*r*_p_ = 0.38±0.08, *r* = 0.58±0.09; n=10), as evident in Fig. 2c (see Fig. 4). The ATR-signal path has two peaks in the sites of the crossing ATR- and SYCP3-signals and is not synchronised with the γH2AFX-signal path (Fig. 2m’).

The RNA Pol II intensively immunostained the whole nucleus, except for the zone where the true part of the sex trivalent is located. In this area the signal is reduced (Fig. 3g–i).

## Discussion

### Specific features of synaptic and recombination behaviour of the XY_1_Y_2_ at pachytene

The sex chromosomes (XY_1_Y_2_) in the common shrew were originally described by Sharman (1956). Later studies of total preparations of SC by light microscopy did not reveal details of XY_1_Y_2_ synapsis at prophase I (Wallace and Searle 1990; Mercer et al. 1992); these were described using electron microscopy (Pack et al. 1993; Narain and Fredga 1997). It was found that the sex trivalent forms an argyrophilic sex body that moves to the nucleus periphery during prophase I. It is interesting that the autosomal part of the sex trivalent is directed into the meiotic nucleus. A similar synapsis of sex chromosomes and the formation of electron-dense material around the true sex chromosome regions within the XY_1_Y_2_ trivalent were identified previously in the bat *Artibeus lituratus* (Solari and Pigozzi 1994) and the deer *Mazama americana* (Aquino et al. 2013). A similar pattern of synapsis in the sex trivalent was also detected in some species of gerbils (Wahrman et al. 1983; Ratomponirina et al. 1986) and in the muntjac; however, in the last case it was difficult to identify clearly the synaptic participants in the absence of electron micrographs (Pathak and Lin 1981).

Desynapsis of the short peritelomeric segment of Y_2_ within the sex trivalent (i.e. chromosome arm *v*: Fig. 1a) has previously been described for several species including the aforementioned bats, deer and gerbils and Pack et al. (1993) already mentioned this phenomenon for the common shrew. From G-banding it looks as if the chromosome arm *v* on the Y_2_ is homologous to an equivalent region on the X chromosome (Fig. 1a). Thus, the desynapsis may be an unusual behaviour of homologous chromatin in proximity to the chromosomal breakpoint of the X-autosome tandem fusion. However, further studies are needed to establish whether the chromosome arm *v* on the Y_2_ is truly homologous to the equivalent region on the X chromosome.

Our data show that each part of the XY_1_Y_2_, the true sex chromosome regions and the translocated parts, displayed one signal of a recombination nodule. A similar pattern of recombination events was revealed previously in common shrew spermatocytes (Borodin et al. 2008) but sometimes these authors visualised two MLH1 signals on the autosomal part of the trivalent, although there usually was a single signal. So, in general features, our results confirmed previous data.

### Chromatin remodelling in the pachytene XY_1_Y_2_

The study of chromatin remodelling of the sex body is possible by immunodetection of specific epigenetic MSCI markers, such as BRCA1, ATR, γH2AFX, SUMO-1 and ubiH2A (Mahadevaiah et al. 2008; Manterola et al. 2009; Page et al. 2012; Sciurano et al. 2012, 2013; Matveevsky et al. 2016; and others). It has previously been found that ATR, γH2AFX, SUMO-1 and ubiH2A play some role in maintaining an inactive form of the chromatin and, in general, in the formation of the sex body (Moens et al. 1999; Mahadevaiah et al. 2001; Rogers et al. 2004; Cao and Yan 2012). In shrew spermatocytes, MSCI starts with the appearance of ATR in the asynaptic region of the X chromosome. After that, the second wave of γH2AFX phosphorylation covers the chromatin associated with the true sex chromosome regions, as shown in our previous work (Matveevsky et al. 2012). Both SUMO-1 and ubiH2A appear simultaneously on the sex trivalent. This picture of MSCI is typical for the XY chromosomes of most mammals, including rodents (Turner 2007; Namekawa and Lee 2009). But the chromatin of the shrew sex trivalent has some distinguishing features, for example, ATR and SUMO-1 are narrowly localised along the axial/lateral elements in both the XY_1_ synaptic region and the asynaptic region within the sex trivalent. We have not seen the spread of the ATR signal into the surrounding chromatin. In contrast, in mice ATR is immunostained along the asynaptic elements with a less intense signal extending into the surrounding chromatin (Turner et al. 2004; Manterola et al. 2009; Fedoriw et al. 2015) and in the mole vole an intense ATR signal surrounds the entire sex bivalent (Matveevsky et al. 2016). SUMO-1 covers the asynaptic region as an extensive cloud in mice (La Salle et al. 2008; Manterola et al. 2009). At the same time, γH2AFX and ubiH2A are as widely distributed over the shrew sex chromatin as in mice and other species (de la Fuente et al. 2007; Sciurano et al. 2012, 2013). Although the chromatin organisation in mammals is universal, a special feature of the epigenetic landscape of sex chromatin has been shown in horses (Baumann et al. 2011) and in human (Metzler-Guillemain et al. 2008). In this case γH2AFX does not cover the chromatin but is localised to the axial elements of the sex bivalent, while ubiH2A is completely absent from the sex body. It is obvious that different epigenetic markers of MSCI may be species-specific features. It is worth noting that we analysed the distribution of the mouse monoclonal ubiH2A, E6C5 clone, while the rabbit monoclonal ubiH2A, D27C4 clone, generates different results (Hasegawa et al. 2015).

The proteins around the true sex chromosome regions of the XY_1_Y_2_ are argentophilic and so the electron-dense cloud is detected around the site of synapsis between X and Y_1_, the unpaired region of the X chromosome, the desynaptic part of the Y_2_ and a short pericentromeric synaptic site between X and Y_2_ (Fig. 1a–d).

**Figure 5.**
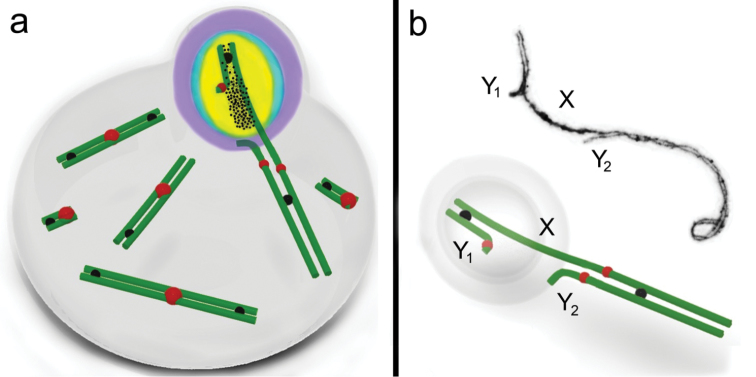
Schematic illustration of male common shrew MSCI. A mid-pachytene spermatocyte (**a**) and a sex (XY_1_Y_2_) trivalent (**b**) of a shrew are shown. An electron micrograph of the sex trivalent is shown at the top of the **b**. The true sex chromosome regions (part of the X and the Y_1_) form a sex body on the periphery of the nucleus. The chromatin of the sex body undergoes reorganisation. MSCI markers have different distributions: SUMO-1 (*yellow*), ATR (*black dots*), ubiH2A (*blue*), γH2AFX (*violet*). ATR is localised on the true sex chromosome regions, and is especially intense on the asynaptic region with a smaller amount where there is synapsis. SUMO-1 and ubiH2A are localised on both the asynaptic and synaptic regions of the true sex chromosome regions. γH2AFX overlays all the true sex chromosome regions and the unpaired part of the Y_2_ axial element. Representative autosomal SCs are shown. MLH1 signals are shown as black balls. The red balls indicate centromeres.

On the basis of immunocytochemistry of MSCI proteins, in this study we suggest a chromatin remodelling model in shrew pachytene spermatocytes (Fig. 5), including two different structural and functional chromatin domains within the sex trivalent: the inactivated chromatin of the true sex chromosome regions and the absence of inactivation in the translocated part. The true sex chromosome regions within the sex trivalent form a macrochromatin domain with both universal and specific features of MSCI, while the translocated part is a typical autosomal chromatin domain. Our interpretations are strongly supported by the distributions of proteins as observed in the preparations, with substantial replication and care in immunostaining and no indications of artefacts that are always a possibility with the spreading technique and efficiency of antibody affinity/sensitivity.

It is worth noting that Pack et al. (1993) assumed, without firm evidence, that the translocated component of the XY_1_Y_2_ in common shrews does not likely undergo inactivation; similar assumptions have been made for other species such as the sex trivalent in the big fruit-eating bat (Solari and Pigozzi 1994). We have been able to use immunological markers to demonstrate that the autosomal component of the sex trivalent (excluding the unpaired part of Y_2_) in the common shrew remains free of the chromatin modifications associated with MSCI.

Thus, our study shows that the shrew sex trivalent (XY_1_Y_2_) has a similar scenario of synapsis and meiotic silencing of unsynapsed chromatin (MSCI) processes as found in the usual sex chromosomes (XY) of male mammals. Apparently, this particular X-autosome translocation does not change the behaviour of the true sex chromosome regions in meiosis and does not affect the process of chromatin transformation at prophase I.

Thus, we may conclude that remodelling of sex chromatin in shrew spermatocytes neatly fits into the MSCI concept.

## Conclusion

A pronounced difference in the structure, behaviour and MSCI of the two parts of the shrew sex trivalent has been revealed on the basis of detailed analysis of the organisation and behaviour of XY_1_Y_2_ at prophase I of meiosis. The ‘head’ part of the trivalent that moves to the periphery of the pachytene nuclei involves the true sex chromosome regions and includes synapsis between the X and Y_1_ chromosomes. The ‘tail’ part involves the region of synapsis between the translocated X and Y_2_ chromosomes. The structure and behaviour of the ‘head’ part (true X region and the Y_1_) including specific MSCI shows patterns which are typical for a male sex bivalent of mammals. At the same time, the ‘tail’ part (the translocated region of the X and the Y_2_) is located among other autosomes and does not differ from them morphologically excluding the fact that this part is attached to the ‘head’ part of the sex trivalent. These dual properties of the ‘head’ and ‘tail’ parts of the XY_1_Y_2_ trivalent in shrew spermatocytes are a notable feature of this system.

It is also noteworthy in this study that we have determined for the first time specific features of MSCI related to the discontinuous distribution of ATR along the SC at the site of synapsis between X and Y_1_ and the distribution limits of SUMO-1 which occurs in the same part of the SC.
